# Characterisation of the Whole Blood mRNA Transcriptome in Holstein-Friesian and Jersey Calves in Response to Gradual Weaning

**DOI:** 10.1371/journal.pone.0159707

**Published:** 2016-08-01

**Authors:** D. Johnston, B. Earley, P. Cormican, D. A. Kenny, M. S. McCabe, A. K. Kelly, M. McGee, S. M. Waters

**Affiliations:** 1 Animal and Bioscience Research Department, Animal & Grassland Research and Innovation Centre, Teagasc, Dunsany, Co. Meath, Ireland; 2 University College Dublin, School of Agriculture Food Science and Veterinary Medicine, Dublin, Belfield, Dublin, 4, Ireland; 3 Livestock Systems Research Department, Animal & Grassland Research and Innovation Centre, Teagasc, Dunsany, Co. Meath, Ireland; Institut national de la santé et de la recherche médicale - Institut Cochin, FRANCE

## Abstract

Weaning of dairy calves is an early life husbandry management practice which involves the changeover from a liquid to a solid feed based diet. The objectives of the study were to use RNA-seq technology to examine the effect of (i) breed and (ii) gradual weaning, on the whole blood mRNA transcriptome of artificially reared Holstein-Friesian and Jersey calves. The calves were gradually weaned over 14 days (day (d) -13 to d 0) and mRNA transcription was examined one day before gradual weaning was initiated (d -14), one day after weaning (d 1), and 8 days after weaning (d 8). On d -14, 550 genes were differentially expressed between Holstein-Friesian and Jersey calves, while there were 490 differentially expressed genes (DEG) identified on d 1, and 411 DEG detected eight days after weaning (P < 0.05; FDR < 0.1). No genes were differentially expressed within breed, in response to gradual weaning (P > 0.05). The pathways, gene ontology terms, and biological functions consistently over-represented among the DEG between Holstein-Friesian and Jersey were associated with the immune response and immune cell signalling, specifically chemotaxis. Decreased transcription of several cytokines, chemokines, immunoglobulin-like genes, phagocytosis-promoting receptors and g-protein coupled receptors suggests decreased monocyte, natural killer cell, and T lymphocyte, chemotaxis and activation in Jersey compared to Holstein-Friesian calves. Knowledge of breed-specific immune responses could facilitate health management practices better tailored towards specific disease sensitivities of Holstein-Friesian and Jersey calves. Gradual weaning did not compromise the welfare of artificially-reared dairy calves, evidenced by the lack of alterations in the expression of any genes in response to gradual weaning.

## Introduction

Weaning of dairy calves is an early life husbandry management practice whereby milk is removed from the calves’ diet and subsequently they derive their nutrient and energy requirements from solid feed alone. Abrupt weaning can act as a multi-factorial stressor in single-suckled beef calves [1–5]. It has been reported to produce an acute pro-inflammatory stress response determined by changes in haematological profiles [4–6], increases in serum concentrations of acute phase proteins [6], up-regulation in expression of genes involved in the pro-inflammatory response [4] and transcriptional alterations in cytokines, chemokines, g-protein-coupled receptors and integrins [2]. However, there is limited published research detailing the immune response to gradual weaning from a liquid based diet in the artificially reared dairy calf. As dairy calves are removed from their dams at birth, gradual weaning of dairy calves may not produce the same stress response associated with abrupt weaning of beef calves. However, gradual weaning of dairy calves takes place months before beef calves are typically weaned from their dams. Consequently, the change-over from a liquid to a solid-feed based diet at such an early stage in rumen development may induce a stress response. One study has reported increases in serum concentrations of both acute phase proteins and cortisol, along with alterations in concentrations of serum inflammatory cytokines (tumour necrosis factor-alpha (TNFα) and interferon-gamma) and an increase in the neutrophil:lymphocyte ratio, demonstrative of a pro-inflammatory response, following gradual weaning in dairy Holstein calves [7]. Furthermore, we have observed differences in haematological profiles and whole blood gene expression of the pro-apoptotic gene *Fas*, in dairy calves in response to gradual weaning [8].

Holstein-Friesian (H-F) and Jersey (J) are the two most predominant dairy breeds used in Ireland [9] and indeed internationally [10]. Body characteristics including birth weight [10] and subsequent growth rate [11] vary greatly between these two breeds. Evidence also exists for transcriptional differences in pooled whole blood between Holstein and J [12]. However, only limited literature suggesting differences in immune responses between H-F and J breeds is currently available. Two studies have reported breed differences in immune responses to infection challenges in cows. Bannerman and co-authors [13] found differences in the onset, magnitude, duration and cessation of acute phase responses and pro-inflammatory cytokine expression between Holstein and J cows after intramammary infection with *Escherichia coli*. Additionally, genes involved in the immune response and antigen presentation and processing differed between Holstein and J cows sub-clinically infected with *Mycobacterium avium* spp. *Paratuberculosis* (MAP) [14].

Alterations in immune responses between calves of these two breeds have also been reported. Serum TNFα was secreted in greater quantities from stimulated mononuclear cells isolated from post-weaned Holstein compared with J calves [15]. Greater bacterial killing and neutrophil oxidative burst activity, were also observed in the whole blood from the post-weaned Holstein calves, following incubation with *Escherichia coli* [15]. Furthermore, calves from J sires had lower total anti-foot-and-mouth disease virus serum antibody titres, in response to three different vaccine strains, than calves from Holstein sires suggesting that the genetic background of calves influences humeral immune responses [16].

RNA-seq has revolutionized transcriptomic studies by providing an efficient approach for transcript discovery, expression analyses, and identification of sequence polymorphisms [17, 18]. It is now being used widely for uncovering multiple facets of the transcriptome to facilitate biological applications [18]. Indeed, alterations in the leukocyte mRNA transcriptome following abrupt weaning in single-suckled beef calves, have been identified using RNA-seq [2]. However, no information is currently available regarding global changes in the mRNA transcriptome in dairy calves, due to gradual weaning. Given that we have previously reported breed differences in haematological profiles and in whole blood expression of several immune genes, during the peri-weaning period [8], the objectives of the current study was to employ an RNA-seq approach to examine differences in global gene expression in whole blood, between artificially reared H-F and J calves, in response to gradual weaning.

## Materials and Methods

All animal procedures performed in this study were conducted under experimental licence from the Irish Department of Health and Children (licence number B100/2869). Protocols were in accordance with the Cruelty to Animals Act (Ireland 1876, as amended by European Communities regulations 2002 and 2005) and the European Community Directive 86/609/EC.

### Animal management

This experiment was conducted as part of a larger study designed to examine changes in haematological profiles and gene expression in response to gradual weaning. The animal model and management has previously been described by Johnston *et al*. [8] and is briefly outlined here. Eight H-F and eight J clinically healthy bull calves with a mean ± s.d. age (days (d)) and bodyweight of 23 ± 7 d, 46 ± 6 kg and 37 ± 8 d, 34 ± 5 kg, respectively, were group housed indoors on sawdust bedded pens at Teagasc, Grange Beef Research Centre from d -56 to d 8 of the study (weaning occurred on d 0). The H-F calves were all sourced from a single farm and the J calves originated from two different farms. Data was missing for the sire of one H-F and one J calf. Six different bulls sired the remaining seven H-F calves. The remaining seven J calves had five different bulls recorded as their sires. At arrival, calves were immunised against infectious bovine rhinotracheitis, parainfluenza-3 virus, bovine respiratory syncytial virus, *Mannheimia haemolytica* serotypes A1 and A6 and *Salmonella dublin* and *Salmonella typhimurium* using Rispoval IBR-Marker live (Pfizer; Co. Cork, Ireland), Bovipast RSP (Intervet; Dublin, Ireland) and Bovivac S (Intervet; Dublin, Ireland) vaccines, respectively.

Calves were individually fed using automatic milk (Vario Powder; Förster-Technik GmbH, Engen, Germany) and concentrate (KFA3-MA3; Förster-Technik GmbH) feeders. The diet offered consisted of a 23% crude protein (CP), 18% lipid milk replacer (Blossom Easymix; Volac, Co. Cavan, Ireland) and concentrate feed (26.5% barley, 25% soya, 15% maize, 12.5% beet pulp, 12.5% soya hulls, 5% molasses, 2.5% minerals, 1% vegetable oil (18.8% CP, 22.4% neutral detergent fibre, 11.06 MJ ME/kg DM)). The pre-weaning, weaning and post-weaning periods were defined as d -56 to d -14, d -13 to d 0 (milk feeding ceased), and d 1 to d 8 respectively. During the pre-weaning period calves were offered 0.8 kg milk replacer (6 litres (l) at 133.33 g/l). The H-F and J calves were offered 1.5 kg and *ad libitum* concentrate pre-weaning, respectively. All calves were offered approximately 400 g straw daily, from a rack within the group pen, throughout the study period. Calves were weaned when consuming 1 kg of concentrate daily for three consecutive days. During the weaning phase milk replacer was gradually reduced from 6 l to 0 l over a 14 d period (d -13 to d 0). The concentrate allocation for the H-F calves was increased to 2 kg/day following gradual weaning. However, as the H-F calves never consumed their maximum allowance of concentrate, they were essentially offered the same amount of concentrate as the J calves.

### Serum sample collection for the zinc sulphate turbidity test

Blood samples were collected via jugular venepuncture into 8.5 ml BD Serum Separator Tube II Advance tubes (BD Vacutainer; Unitech, Dublin, Ireland) on the day of arrival of the calves to Teagasc, Grange Beef Research Centre. Serum was harvested from these samples and stored at -20°C. The zinc sulphate turbidity (ZST) (proxy for immunoglobulin status) analysis was subsequently performed at 20°C and turbidity measurements were obtained at 520 nm using a spectrophotometer [19].

### Statistical analysis of zinc sulphate turbidity test data

The data were examined for adherence to a normal distribution (PROC UNIVARIARTE, SAS v 9.3). Zinc sulphate turbidity data were analysed using mixed models ANOVA (PROC MIXED, SAS v 9.3). Differences between the means were tested using the PDIFF option within the MIXED procedure of SAS. Means were considered statistically significantly different at a probability level of P < 0.05.

### Whole blood sample collection for RNA-seq analysis

**Fig 1:**
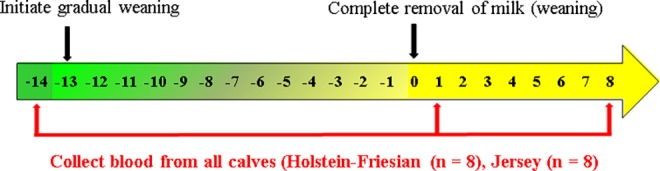
Illustration of the animal model, specifically the days relative to weaning and the days the calves were blood-sampled relative to weaning. The arrow contains day numbers relative to weaning. The first black arrow shows that gradual weaning was initiated on day -13. The second black arrow represents weaning when milk was removed from the diet on day 0. The red arrows show the days the calves were blood sampled relative to weaning, days -14, 1 and 8.

Relative to weaning (d 0), on d -14, d 1, and d 8, blood samples (3 mL) were collected via jugular venepuncture into Tempus^TM^ blood RNA tubes containing RNA stabilisation solution (Applied Biosystems; Foster City, California, USA) (Fig 1). Immediately after blood collection, the Tempus blood RNA Tubes were shaken vigorously by hand for 20 seconds and were subsequently stored at -80°C until analysis.

### RNA extraction from whole blood

Total RNA was extracted from whole blood using the Tempus^TM^ Spin RNA Isolation Reagent Kit (Applied Biosystems; Foster City, California, USA), according to manufacturer’s instructions. AbsoluteRNA Wash Solution (Applied Biosystems; Foster City, California, USA) was used for the DNase step. The quantity of the extracted RNA was determined by measuring the absorbance at 260 nm with a Nanodrop spectrophotometer (NanoDrop technologies; Wilmington, DE, USA). The quality of the extracted RNA was examined with the Agilent 2100 Bioanalyser (Agilent Technologies Ireland Ltd; Dublin, Ireland) using the RNA 6000 Nano LabChip kit (Agilent Technologies Ireland Ltd; Dublin, Ireland). Samples had an RNA Integrity Number (RIN) of mean (± s.d.) 9.6 (± 0.29).

### RNA-seq library preparation

Forty-eight individual libraries were prepared from a starting material of 1000 ng high quality total RNA using the Illumina TruSeq RNA Sample Preparation Kit v2 (Illumina Inc; San Diego, CA, USA), according to manufacturer’s instructions. Library preparation initially involved purification of mRNA from total RNA and fragmentation of the mRNA. SuperScript II Reverse Transcriptase (Applied Biosystems; Foster City, California, USA) was subsequently used for the synthesis of the first strand of the cDNA. The second strand of cDNA was synthesised using components of the Illumina TruSeq RNA Sample Preparation Kit v2. Adaptors were ligated to the cDNA samples in a manner which allowed random pooling of libraries and limited technical variation in the experiment. The cDNA fragments containing the adapters were enriched by PCR. Validation of individual libraries was performed with the Agilent Bioanalyser 2100 using the DNA 1000 Nano LapChip kit (Agilent Technologies Ireland Ltd; Dublin, Ireland). It confirmed that library fragment sizes were ~260 base pairs (bp). Libraries were quantified with a Qubit Fluorometer. Equal quantities of individual libraries were pooled based on their respective sample-specific-6 bp adaptors and sent to Clinical Genomics (Toronto, Canada), for RNA sequencing on an Illumina HiSeq 2500 with four libraries per lane. Forty million 76 bp paired end reads were sequenced per library.

### Alignment of sequenced reads to the bovine genome and differential gene expression analysis

**Fig 2:**
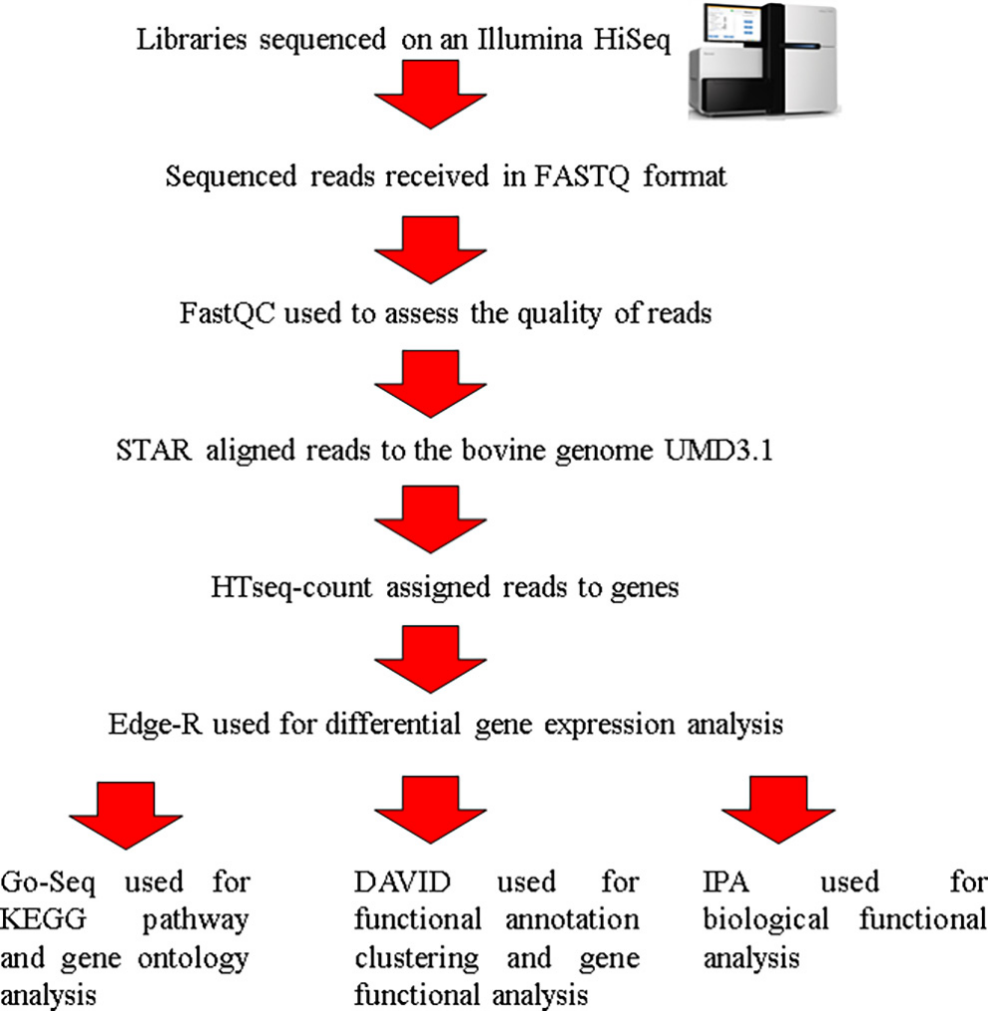
Illustration of the bioinformatic pipeline utilised for RNA-seq data analysis. Paired-end sequencing was performed on the Illumina HiSeq, FastQC was used to assess sequencing quality, reads were aligned to the bovine genome with Spliced Transcripts Alignment to a Reference (STAR), counts were assigned to genes with HTseq-count, Edge-R was used to calculate differential gene expression, Kyoto encyclopaedia of genes and genomes (KEGG) pathway and gene ontology analysis were performed in GoSeq, functional annotation clustering and gene functional analysis was performed with Database for Annotation, Visualization and Integrated Discovery (DAVID), biological functional analysis was performed with Ingenuity Pathway Analysis (IPA) downstream effects.

Raw sequence reads were received in FASTQ format and assessed for quality using FastQC (version 0.10.0) (http://www.bioinformatics.babraham.ac.uk/projects/fastqc/) (Fig 2). All reads passed the basic quality statistics. Reads were aligned to the bovine genome UMD3.1 using the Spliced Transcripts Alignment to a Reference (STAR) aligner [20] (Fig 2). Reads were only aligned if they contained fewer than two mismatches with the reference genome and uniquely mapped to the reference genome. All retained reads were output in SAM format and subsequently converted to BAM format, sorted, and converted back to SAM format using SAMtools [21]. Read counts were generated using HTseq-count to convert aligned reads into counts per gene using the union model and the Ensembl UMD3.1 annotation of the bovine genome [22] (Fig 2). Differential gene expression was determined using the R (version 3.0.1 (2013-05-16)) Bioconductor package EdgeR (version 3.2.4) [23] (Fig 2), which accounts for biological and technical variation by modelled data as a negative binomial distribution using a generalisation of the Poisson distribution model. To filter out lowly expressed genes, genes with less than one count per million in at least eight samples, were discarded from the analysis. Data were normalised across libraries using the trimmed mean of M-values normalization method [24]. The quantile-adjusted conditional maximum likelihood (qCML) common dispersion and the qCML tagwise dispersions were used to estimate dispersion. Exact tests were used for the detection of differentially expressed genes (DEG) between time-points within breed and between breeds at each time-point. Genes with a Benjamini-Hochberg false discovery rate (FDR) of 10% and a fold change of ≥ 1.5 were considered differentially expressed. All sequence data produced in this study has been deposited to NCBI GEO repository and are available through series accession number GSE76841.

### Pathway analysis

Pathway analysis was performed using GOSeq (version 1.12.0) [25], with the Kyoto encyclopaedia of genes and genomes (KEGG) database [26] (Fig 2), on statistically significant (P < 0.05) DEG between breeds, at each time-point, with a FDR of 10% and a fold change of > 1.5. Differences in gene length can bias pathway and functional analyses. There is more power to detect longer genes as differentially expressed in RNA-seq experiments as the total number of reads for a gene is proportional to its expression multiplied by its length [25]. GOSeq took gene length into account as it calculated the Probability Weighting Function for each gene and used it to quantify the likelihood of a gene being differentially expressed based on its length alone, and subsequently corrected the analysis for gene length bias. Pathways which were represented significantly more among the DEG, than would be expected due to random chance, were identified using the GoSeq default method “Wallenius” by the Wallenius non-central hypergeometric distribution. A 10% FDR cut-off was implemented on the results of the pathway analysis using Bioconductor’s qvalue package (version 1.34.0) [27].

### Gene ontology analysis

Gene ontology analysis was performed using GOSeq (version 1.12.0) [25] (Fig 2), on statistically significant (P < 0.05) DEG between breeds, at each time-point, with an FDR of 10% and a fold change of > 1.5. As in GOSeq/KEGG pathway analysis, GoSeq corrected for gene length bias and identified ontological terms which were represented significantly more than would be expected due to random chance. Gene ontological terms were identified as over-represented when they had a P value of less than 0.05 and an FDR of 10%.

### Functional annotation clustering and gene grouping analysis

The DEG between breeds, at each time-point, with an FDR of 10% and a fold change of > 1.5, were analysed for functional annotation clustering and gene functional classification using the Database for Annotation, Visualization and Integrated Discovery (DAVID) (http://david.abcc.ncifcrf.gov/tools.jsp) [28, 29] (Fig 2). The background genes consisted of all the expressed genes for the specific time-point being analysed. The annotation categories for DAVID analysis were set to have: a Kappa similarity of 4; a similarity threshold of 0.35; an initial group membership of 4; a final group membership of 4 and a multiple linkage threshold of 0.5. The enrichment P-values of each annotation term were derived from a modified Fisher’s exact test called EASE score. The group enrichment score of each functional annotation cluster or gene functional classification group was calculated from the geometric mean (in -log scale) of all the enrichment P-values in each annotation term within each cluster and was used to determine the significance of results from the functional annotation clustering and gene functional classification analyses. A group enrichment score cut-off of 1.3 (EASE = 0.05) was applied in the present study.

### Biological functional analysis

To examine the cellular and molecular functions, the RNA-seq data were further analysed using Ingenuity Pathway Analysis (IPA) downstream effects (v. 21249400, Ingenuity Systems, Mountain View, CA; http://www.ingenuity.com), according to the manufacturer’s instructions (Fig 2). Within IPA, the Fisher’s exact test corrected by the Benjamini-Hochberg test was used for the identification of over-represented molecular and cellular functions with a FDR of 10%, from DEG between breeds, at each time-point, with an FDR of 10% and a fold change of > 1.5. Additionally, IPA’s regulation z-score algorithm, which predicts increases or decreases in functions based on directional changes in the DEG and expectations derived from the literature, was used to predict differences in the over-represented cellular and molecular functions. IPA software considered cellular and molecular functions with a regulation z-score value of ≥ 2.0 to be significantly increased and cellular and molecular functions with a regulation z-score value of ≤ -2.0 to be significantly decreased.

## Results

### Passive immunity

The J calves had greater maternally derived passive immunity (Lsmeans (s.e.m.)) (19.1 (0.97) units) than H-F calves (15.9 (0.97) units) (P < 0.05).

### Differentially expressed genes

**Fig 3:**
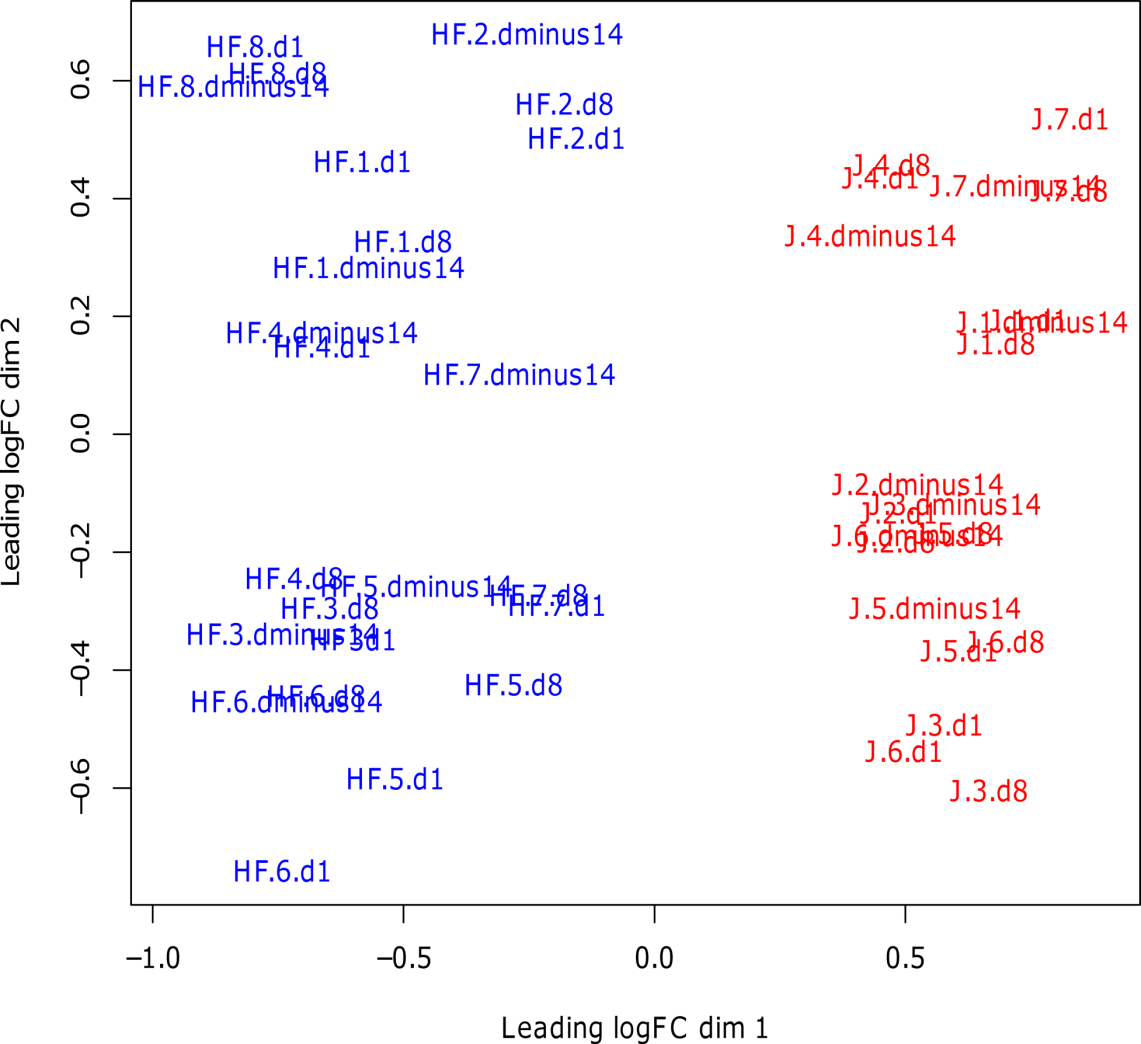
Multidimensional scaling plot which shows the measured similarity of the samples in 2-dimensions. The first letters represents the breed of each calf (HF = Holstein-Friesian, J = Jersey), the following number represents the ID number (1–8 for Holstein-Friesian and 1–7 for Jersey) and the final text and numbers represents the time-point relative to weaning (dminus14 = day -14, d1 = day 1, d8 = day 8). The samples labelled in blue are Holstein-Friesian and the samples labelled in red are Jersey.

The samples from one J calf contained outliers, and consequently, this calf was removed from all analyses. Using Edge-R, a multi-dimensional scaling (MDS) plot was produced which measured the similarity of the samples and projected this measure into 2-dimensions. It illustrated a separation between the two breeds but no separation occurring between time-points (Fig 3).

**Fig 4:**
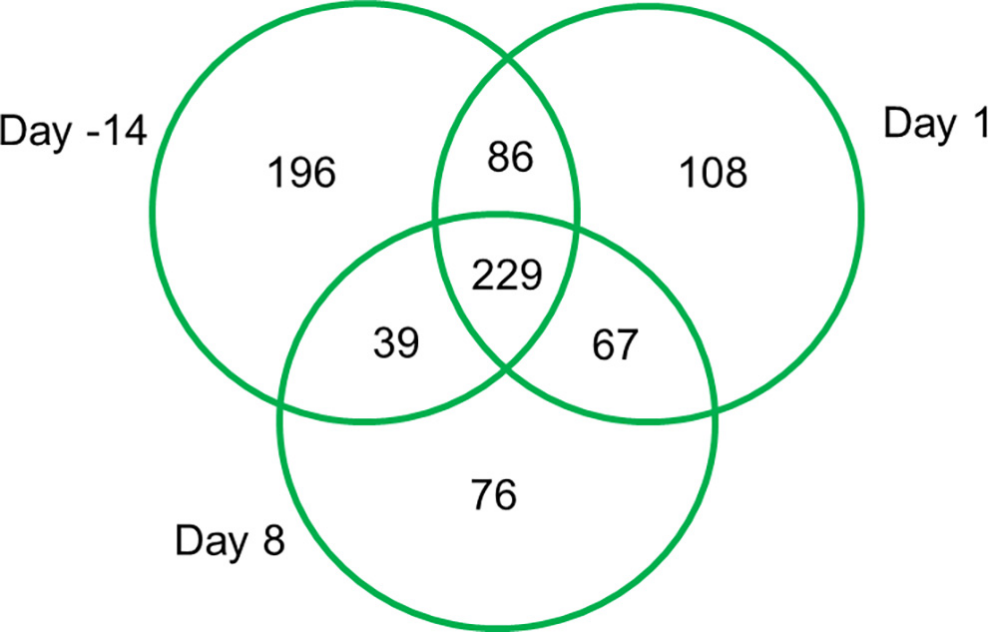
Venn diagram of the DEG between Holstein-Friesian and Jersey calves at each time-point. Each circle represents a time-point relative to weaning (i.e. day -14, day 1 and day 8).

Gradual weaning had no effect on gene expression as there were no DEG within breed between time-points (P > 0.05). However, gene expression differed between the two breeds at each time-point (P < 0.05). There were 550 DEG (P < 0.05; FDR < 0.1; fold change > 1.0) between H-F and J at d -14 (S1 Table). Two hundred and fourteen of these genes had increased transcription while 337 genes were transcriptionally decreased, in J relative to H-F calves. Additionally, there were 490 DEG (P < 0.05; FDR < 0.1; fold change > 1.5) between H-F and J at d 1 (S2 Table) with 242 of these genes transcriptionally increased and 248 transcriptionally decreased in J compared with H-F. There were 411 DEG (P < 0.05; FDR < 0.1; fold change > 1.5) between H-F and J at d 8 (S3 Table). One hundred and fifty four of the genes had increased expression and 257 of the genes had decreased expression in J relative to H-F. The majority of the DEG (229) between breeds were common to all time-points (Fig 4).

### Pathway analysis of differentially expressed genes between breeds

Results of KEGG pathway analysis using GoSeq, after the implementation of an FDR of 10% using qvalue, indicated that nine KEGG pathways were over-represented among the DEG between H-F and J calves on d -14, five on d 1 and five on d 8 (P < 0.01; Q ≤ 0.1) (Table 1). Two KEGG pathways, the cytokine-cytokine receptor interaction pathway (KEGG I.D. = bta04060) and the neuroactive ligand-receptor interaction pathway (KEGG I.D. = bta04080) were over-represented among the DEG between H-F and J calves at each time-point (d) (P<0.01; Q ≤ 0.1) (Table 2). The majority of DEG within these pathways were transcriptionally decreased in J relative to H-F calves (Table 2). Within the cytokine-cytokine receptor interaction pathway, there was a consistent decrease in the transcription of eight genes and increased transcription of two genes in J relative to H-F calves, at all time-points relative to weaning (Table 2). Five genes consistently showed decreased transcription and one gene was transcriptionally enhanced, in J relative to H-F calves, within the neuroactive ligand-receptor interaction pathway, at all time-points relative to weaning Table 2).

**Table 1:** Over-represented KEGG pathways which were returned by GOseq analysis of genes differentially expressed between Holstein-Friesian and Jersey calves at each time-point relative to weaning (P < 0.05; Q < 0.1).

Rank	Day -14	Day 1	Day 8
**1**	Cytokine-cytokine receptor interaction	Lysine biosynthesis	Cytokine-cytokine receptor interaction
**2**	Phagosome	Arachidonic acid metabolism	Neuroactive ligand-receptor interaction
**3**	Staphylococcus aureus infection	Staphylococcus aureus infection	Phagosome
**4**	Cell adhesion molecules (CAMs)	Cytokine-cytokine receptor interaction	ECM-receptor interaction
**5**	Basal cell carcinoma	Neuroactive ligand-receptor interaction	Focal adhesion
**6**	Melanogenesis		
**7**	Protein digestion and absorption		
**8**	ECM-receptor interaction		
**9**	Neuroactive ligand-receptor interaction		

Rank = significance relative position (1 is the most significant, 9 is the least significant)

Day = day relative to weaning

**Table 2:** Over-represented KEGG pathways returned by GOseq analysis of genes differentially expressed between Holstein-Friesian and Jersey at all three different time points relative to weaning.

KEGG Pathway	Time-points	Transcription increased	Transcription decreased
**Cytokine-cytokine receptor interaction (bta04060)**	d -14	*TNFRSF13C*, *CXCR5*, *TNFRSF13C*, *PDGFA*, *IFNB1*	*CXCL10*, *MPL*, *IL1R2*, *PDGFRA*, *CCL5*, *CXCL4*, *IL23R*, *IL13RA1*, *CCL3* (ensemble ID = ENSBTAG00000025250), *TNFSF12*, *CXCL16*, *IL18RAP*, *TNFSF13B*, *TNFSF9*, *IL15RA*, uncharacterised protein (ensemble ID = ENSBTAG00000011563)
	d 1	*TNFRSF13C*, *PDGFA*, *IFNB1*	*CXCL10*, *KIT*, *MPL*, *IL1R2*, *PDGFRA*, *CCL5*, *CXCL4*, *TNFSF12*, *PPBP*, *IL18RAP*, two genes annotated *CCL3* (ensemble IDs = ENSBTAG00000025250 and ENSBTAG00000003289)
	d 8	*MET*, *PDGFA*, *IFNB1*	*IL1B*, *MPL*, *IL1R2*, *PDGFRA*, *CCL5*, *CXCL4*, *FLT3*, *TNFSF12*, *IL18RAP*, *CXCR2*, two genes annotated *CCL3* (ensemble IDs = ENSBTAG00000025250 and ENSBTAG00000003289)
**Neuroactive ligand-receptor interaction (bta04080)**	d -14	*HTR6*, *GH1*	*F2RL2*, *CALCRL*, *LTB4R*, *C3AR1*, *P2RY14*, *PRSS2*, *GZMA*, *ADORA3*, *P2RX1*
	d 1	*GIPR*, *HTR6*	*CALCRL*, *LTB4R*, *C3AR1*, *HRH4*, *C5AR1*, *P2RY14*, *PRSS2*, *GZMA*
	d 8	*S1PR3*, *HTR6*	*ADRB1*, *S1PR5*, *CALCRL*, *LTB4R*, *C5AR1*, *P2RY14*, *PRSS2*, *GZMA*

Time-points = days relative to weaning; Transcription increased = increased transcription in Jersey relative to Holstein-Friesian calves; Transcription decreased = decreased transcription in Jersey relative to Holstein-Friesian calves

### Gene ontology analysis of differentially expressed genes between breeds

Six molecular functions, five biological processes and six cellular components were over-represented among the DEG between breeds in the GoSeq analysis on d -14 (P < 0.05; FDR < 0.1) (S4 Table). Twenty molecular functions, 11 biological processes and seven cellular components were over-represented among the DEG between breeds in the GoSeq analysis on d 1, while on d 8, 13 molecular functions, 41 biological processes and eight cellular components were over-represented among the DEG between breeds (P < 0.05; FDR < 0.1) (S4 Table). Three molecular functions, one biological process and four cellular components were over-represented among the DEG between breeds, common to every time-point (Table 3).

**Table 3:** Over-represented ontological terms returned by GOseq analysis of genes differentially expressed between Holstein-Friesian and Jersey calves at all three different time points relative to weaning.

Molecular Function		Biological Process		Cellular Component	
GO ID	Term	GO ID	Term	GO ID	Term
**GO:0008009**	chemokine activity	GO:0033002	muscle cell proliferation	GO:0005576	extracellular region
**GO:0042379**	chemokine receptor binding			GO:0005615	extracellular space
**GO:0005125**	cytokine activity			GO:0044421	extracellular region part
				GO:0032994	protein-lipid complex
				GO:0034358	plasma lipoprotein particle

GO = gene ontology

### Functional annotation clustering of differentially expressed genes between breeds

Functional annotation clusters were identified as enriched among the DEG between breeds (group enrichment score > 1.3) on day -14 (S5 Table), day 1 (S6 Table) and day 8 (S7 Table), relative to weaning. Overall, the clustered functions which were enriched among the DEG between breeds at all time-points relative to weaning were related to cell signalling and immune responses. Clustered functions included cytokine and chemokine activity, C-type lectin activity, immunoglobulin like and MH1 class receptor activity, g-protein coupled signalling and cell membrane and extracellular signalling.

### Gene functional classification grouping of differentially expressed genes between breeds

Gene functional classification groups were identified as enriched among the DEG between breeds (group enrichment score > 1.3) on d -14 (S8 Table), d 1 (S9 Table) and d 8 (S10 Table), relative to weaning.

The gene groups which were consistently enriched among the DEG between breeds at all time-points relative to weaning, included: g-protein coupled receptors; chemokines; lectin receptors; complement receptors and interleukin receptor accessary proteins.

### Biological function analysis of differentially expressed genes between breeds

**Fig 5:**
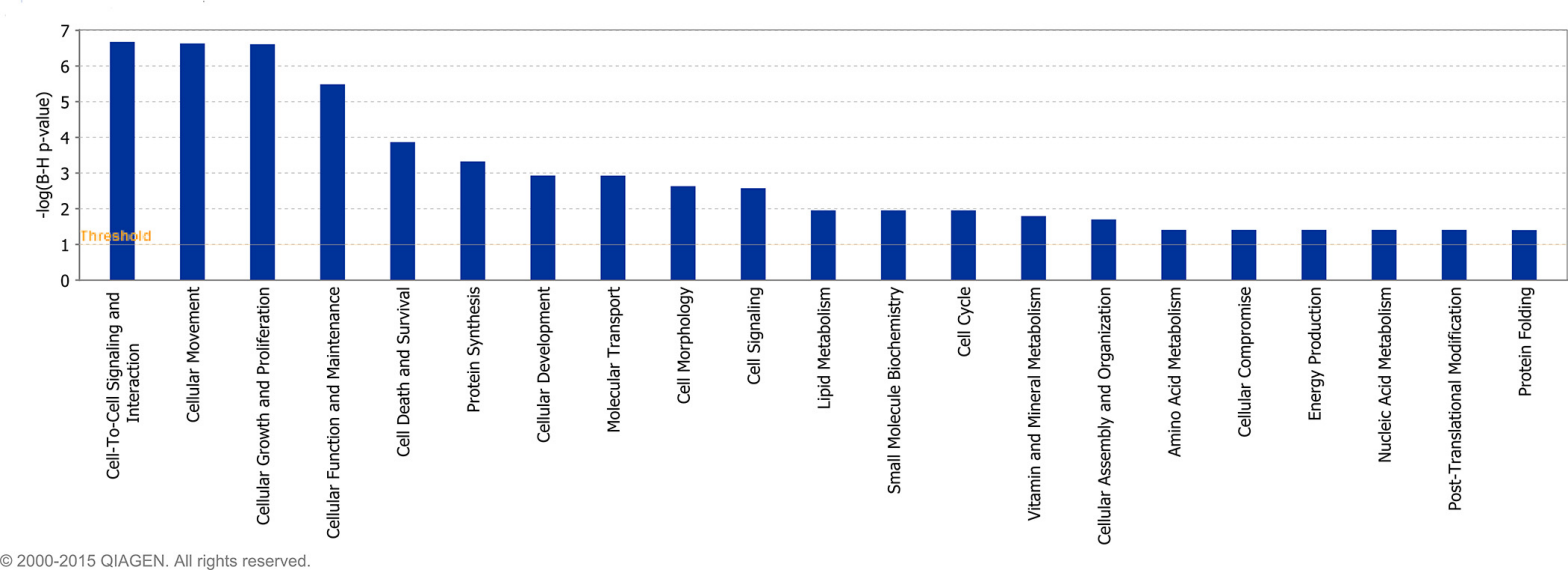
Over-represented molecular and cellular functions returned by Ingenuity Pathway Analysis of genes differentially expressed between Holstein-Friesian and Jersey calves on day -14 relative to weaning (P < 0.05; FDR < 0.1).

**Fig 6:**
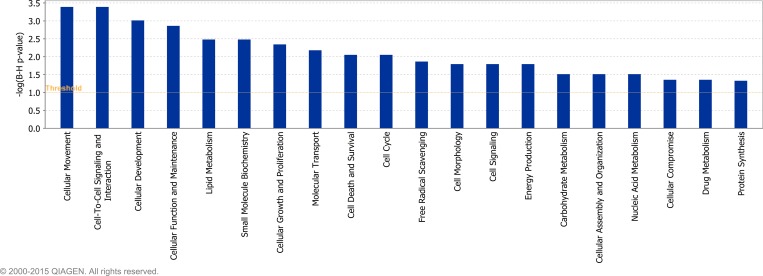
Over-represented molecular and cellular functions returned by Ingenuity Pathway Analysis of genes differentially expressed between Holstein-Friesian and Jersey calves on day 1 relative to weaning (P < 0.05; FDR < 0.1).

**Fig 7:**
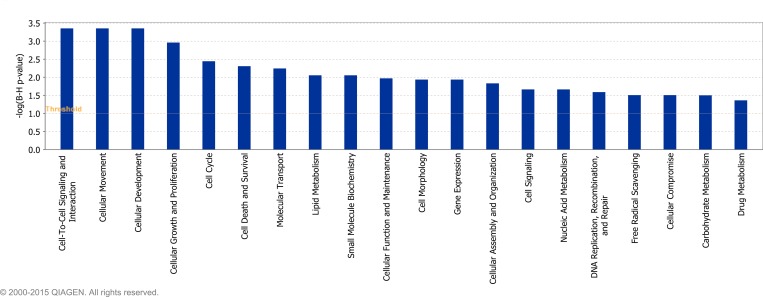
Over-represented molecular and cellular functions returned by Ingenuity Pathway Analysis of genes differentially expressed between Holstein-Friesian and Jersey calves on day 8 relative to weaning (P < 0.05; FDR < 0.1).

Twenty-five (Fig 5), twenty-four (Fig 6) and twenty (Fig 7) molecular and cellular functional categories were found to be over-represented among the DEG between the breeds, using the IPA molecular and cellular function category analysis (P < 0.05; FDR < 0.1), on d -14, d 1 and d 8, respectively. Within these categories, IPA predicted the activation state (increased or decreased) of several functions in J relative to H-F calves on d -14 (S11 Table), d 1 (S12 Table) and d 8 (S13 Table), relative to weaning. Functions including leukocyte migration and chemotaxis, cell engulfment, and cell binding were consistently predicted to be decreased in J relative to H-F calves across all time-points relative to weaning (Table 4).

**Table 4:** Predicted activation states of biological functions in Jersey relative to Holstein-Friesian at all three different time points relative to weaning, returned by Ingenuity Pathway Analysis.

Functions annotation	Predicted activation state	Genes with increased transcription in Jersey calves	Genes with decreased transcription in Jersey calves
**Cell-to-cell signalling and interaction category:**			
**Binding of cells**	Decreased	*BTLA*, *CLGN*, *ERBB2*,	CALCRL, CCL3*, CCL5, CDH5, CLEC10A, IL18RAP, IL1R2, LPL, MSR1, PF4, PRSS2, PTX3, TGM2
**Adhesion of blood cells**	Decreased		CCL3*, CCL5, CLEC10A, CLU, LTB4R, MSR1, PF4, PTX3, TGM2, TNFSF12
**Cellular movement category:**			
**Chemotaxis of cells**	Decreased	*BMP4*, *ERBB2*, *PROX1*	AIF1, CCL3*, CCL5, CDKN1A, CLU, LTB4R, NLRP12, NRP1, PDGFRA, PF4
**Cell movement of blood cells**	Decreased	*BACH2*, *BMP4*, *BTLA*, *ERBB2*, *JAM2*, *PROX1*	AIF1, CCL3*, CCL5, CDKN1A, CLU, EGR2, IDO1, LTB4R, MSR1, NLRP12, PDGFRA, PF4, PLXND1, PTX3, SIGLEC8, TGM2, TNFSF12
**Cell movement of leukocytes**	Decreased	*BACH2*, *ERBB2*, *JAM2*	AIF1, CCL3*, CCL5, CDKN1A, EGR2, IDO1, LTB4R, MSR1, NLRP12, PF4, PLXND1, PTX3, SIGLEC8, TGM2, TNFSF12
**Cell movement of antigen presenting cells**	Decreased	*ERBB2*	CCL3*, CCL5, CDKN1A, NLRP12, PF4, PLXND1, PTX3, TNFSF12
**Cell movement of myeloid cells**	Decreased	*ERBB2*	AIF1, CCL3*, CCL5, CDKN1A, LTB4R, MSR1, NLRP12, PF4, PLXND1, PTX3, SIGLEC8, TGM2, TNFSF12
**Cell movement of phagocytes**	Decreased	*ERBB2*	AIF1, CCL3*, CCL5, CDKN1A, LTB4R, NLRP12, PF4, PLXND1, PTX3, TNFSF12
**Chemotaxis of phagocytes**	Decreased	*ERBB2*	AIF1, CCL3*, CCL5, CDKN1A, LTB4R, NLRP12, PF4,
**Chemotaxis of myeloid cells**	Decreased	*ERBB2*	AIF1, CCL3*, CCL5, CDKN1A, LTB4R, NLRP12, PF4
**Chemotaxis of neutrophils**	Decreased		CCL3*, CCL5, LTB4R, NLRP12, PF4
**Cell movement of neutrophils**	Decreased	*ERBB2*	CCL3*, CCL5, LTB4R, NLRP12, PF4, PTX3
**Leukocyte migration**	Decreased	*BACH2*, *BTLA*, *ERBB2*, *JAM2*, *PROX1*	AIF1, CCL3*, CCL5, CDKN1A, CLU, EGR2, IDO1, LTB4R, MSR1, NLRP12, PDGFRA, PF4, PLXND1, PTX3, SIGLEC8, TGM2, TNFSF12
**Cellular function and maintenance category:**			
**Engulfment of cells**	Decreased	*ERBB2*,	CCL3*, CCL5, FCN1, MSR1, PF4, PTX3, TGM2

*CCL3 = ensemble ID: ENSBTAG00000025250

## Discussion

This is the first study to examine the whole blood mRNA transcriptomic response to weaning in two breeds of dairy calf. Gradual weaning did not induce a systemic immune response in either H-F or J calves as no differentially expressed genes were detected within breed between time-points by RNA-seq analysis. These results are consistent with the observation that the typical neutrophilia and lymphopenia haematological responses associated with stress, were absent in these calves, following gradual weaning [8]. Therefore, the gradual weaning practiced in this study is a welfare friendly method of weaning dairy calves. However, different types of gradual weaning management practices may not be as welfare friendly. For example, Kim, *et al*. [7] initiated gradual weaning early, when calves were 29 to 30 days of age, and completely weaned calves from milk at the age of 42 days. At this relatively young age, the calves may not have been consuming sufficient amounts of concentrate for appropriate rumen maturation before removal of milk from the diet, and consequently, haematological profiles associated with a pro-inflammatory stress response, increases in concentrations in serum of both acute phase proteins and cortisol, and alterations in concentrations of serum inflammatory cytokines, were observed [7].

Although, in the current study, there were no DEG within breed in response to gradual weaning, there were many genes differentially expressed between the two breeds at each time-point relative to weaning. This is in agreement with Huang and colleagues who discovered differential gene expression in pooled whole blood between Holstein and J cows [12] and with Verschoor and colleagues who identified differential gene expression in peripheral blood mononuclear cells from MAP infected Holstein and J cows [14]. Interestingly, many of the DEG in the present study were associated with cell signalling and the immune response and were consistently differently expressed between the H-F and J calves during the peri-weaning period. Cytokine and chemokine signalling and activity, including g-protein coupled receptor interaction and the induction of leucocyte chemotaxis and phagocytosis, were identified as key pathways and functions transcriptionally altered between the two breeds. Interestingly, these pathways and functions were consistently enriched with all the bioinformatics tools employed, GoSeq (which accounted for gene length), DAVID which did not account for gene length but placed similar functions and genes into groups and IPA which predicted functional differences based on global gene expression alterations. This suggests that breed influences immune responses as cytokines and chemokines have functional roles in homeostasis, inflammation, and disease modulation [30].

Chemokines are an integral part of the immune response to pathogens, as following binding to g-protein coupled receptors [31, 32], and glycosaminoglycans (GAG)s such as heparin sulphate, they provide the fixed directional cues to leukocytes to direct their migration into target tissues [33]. Jersey calves had consistently decreased transcription of the *CCL5* and *CCL3*, chemokines, compared to H-F calves, at all time-points relative to weaning. Interestingly, both CCL5 and CCL3 are primarily inflammatory monocyte chemotactic chemokines [34]. Monocytes are an important component of the innate immune response to infection as they are the precursors of dendritic cells and macrophages and their recruitment to sites of infection is necessary for control and clearance of viral, bacterial, fungal and protozoan pathogens [34]. However, CCL5 and CCL3 also contribute to other aspects of the innate immune response, including natural killer cell chemotaxis and cytotoxicity [35]. As well as participating in the innate immune response, CCL3 and CCL5 also function in cell-mediated adaptive immunity as they both attract T lymphocytes to sites of infection and they preferentially recruit T helper (Th) type 1 differentiated cells [36].

In addition to chemokines, many cytokines which are consistently transcriptionally decreased in J relative to H-F calves, at all time-points relative to weaning, are also involved in innate immune cell activation and chemotaxis. The cytokines CXCL4 (also known as PF4), and TNFSF12 participate in monocyte induced immune responses, including chemotaxis, phagocytosis, survival, differentiation into macrophages and pro-inflammatory cytokine secretion [37, 38]. The *IL18RAP* gene codes for an accessory subunit of the heterodimeric receptor for interleukin 18 and it enhances natural killer cell cytotoxicity, promotes IFNy production and neutrophil activation [39]. The *MPL* and *PDGFRA* genes play an important role in the vascular response to injury and wound healing as MPL regulates the production of platelets [40] and PDGFRA is a chemoattractant receptor for mesenchymal cells [41, 42]. However, as the expression of the gene coding for the PDGFRA ligand, *PDGFA* showed increased transcription in J relative to H-F calves, it is impossible to speculate whether mesenchymal cell chemotaxis is increased or decreased in J relative to H-F calves.

Furthermore, genes with immunological functions which are not part of the cytokine or chemokine families but play important roles in the host immune response to infection were found to be transcriptionally decreased in J relative to H-F calves. These include genes that are part of the neuroactive ligand-receptor interaction pathway, c-type lectin and immunoglobulin-like functional groups and genes with chemotactic- and phagocytosis-promoting functions. For example, there was decreased transcription of *LTB4R*, the gene coding for the leukotriene B4 receptor which functions in the induction of chemotaxis following ligand binding, in J relative to H-F, at all time-points relative to weaning. It is an important mediator of the inflammatory immune response as it is involved in the recruitment of pro-inflammatory cells (including neutrophils [43] macrophages, eosinophils, mast cells, dendritic cells and effector T cells [44]) to sites of infection [45]. Additionally, genes involved in the cellular killing activity of natural killer and cytotoxic T cells were transcriptionally decreased in Jersey calves at all time-points. For example, granzymes A and M, which belong to a family of granule serine proteases found in the cytotoxic granules of these immune cell types [46], were transcriptionally decreased in J relative to H-F calves. These proteases play a role in antiviral activity through induction of cell death following introduction into target cells [47, 48]. Moreover, killer cell immunoglobulin-like receptor, two domains, short cytoplasmic tail *(KIR2SL1)*, which is expressed on the surface of natural killer cells, and activates natural killer cell activity following binding to certain MHC complex class 1 allotypes [49], showed decreased transcription in J relative to H-F calves.

Genes coding for phagocytosis-promoting receptors including the C-type lectin receptor DC-SIGN and the scavenger receptor MSR1 were also consistently down-regulated in J relative to H-F calves. DC-SIGN plays an important role in dendritic cell immune regulation as it is a pattern recognition and adhesion receptor which can induce dendritic cell adhesion and migration to infection sites, inflammatory responses and T cell activation [50]. MSR1, a transmembrane cell surface glycoprotein expressed on macrophages and dendritic cells can bind bacterial surface proteins such as LPS and LTA, bacterial CpG and double-stranded RNA and can mediate non-opsonic phagocytosis of bacterial pathogens [51].

The consistent decreased transcription of *CCL3*, *CCL5*, *CXCL4*, *TNFSF12*, *IL18RAP*, *LTB4R*, *GZMA*, *GZMM*, *KIR2SL1*, *MSR1* and *DC-SIGN* in J relative to H-F calves suggests functional decreases in leukocyte chemotaxis (especially monocyte chemotaxis) and phagocytic activity along with the natural killer cell and Th 1 cell-mediated responses. Furthermore, several additional cytokines with similar related functions were decreased in J relative to H-F calves on at least one day relative to weaning. Jersey calves had reduced transcription of *CXCL10* (also known as *IP-10*) compared with H-F calves, on days -14 and 1, relative to weaning. The CXCL10 protein is an IFNγ inducible inflammatory chemokine associated with a Th 1 phenotype and it plays an important role in leukocyte recruitment (especially monocytes and T lymphocytes) to inflammatory sites [52]. Expression of *IL23R* was also reduced in J relative to H-F calves on d -14. It codes for a receptor subunit for IL-23 which promotes proliferation of T cells, stimulates IFNγ production by T cells and promotes Th 1 differentiation [53, 54]. Additionally, transcription of *IL1B* was decreased in J relative to H-F calves on d 8. It codes for a pro-inflammatory cytokine produced mainly by monocytes and macrophages and is involved in the enhancement of secretion of other pro-inflammatory cytokines, fever induction, histamine release from basophils, macrophage phagocytosis and neutrophil survival, adhesion and oxidative burst activation and survival, differentiation and proliferation of T cells [55].

Interestingly, the expression of two genes involved in the pro-inflammatory immune response was consistently increased in J relative to H-F calves, at all time-points relative to weaning. Jersey calves had increased levels of transcription of *IL1R2* which codes for a decoy receptor for IL1 and modulates pro-inflammatory immune responses [56] and of *IFNB1* which is involved in the bovine protective immune response against specific viruses including RNA viruses [57]. Therefore, both breeds have different levels of transcription for important genes functioning in immune responses and likely have divergent susceptibilities and immune responses for specific pathogen types. Indeed many of the genes which are differentially expressed between these two breeds have been implicated in the immune response to several bovine diseases. The *CCL5* chemokine has been shown to play a role in the inflammatory response to bovine respiratory syncytial virus [58], Alcelaphine herpesvirus 1 [59], mastitis [60–63], bovine tuberculosis [64] and parasitic protozoan infections with *Toxoplasma gondii* [65], *Neospora canium* [65] and *Eimera bovis* [65]. Up-regulation of *CCL3* has previously been associated with the inflammatory response to mastitis [62], bovine tuberculosis [64, 66], viruses including bovine respiratory syncytial virus [58], Alcelaphine herpesvirus 1 [59], resistance to bovine nematode infections [67] and a protective response from vaccinated cattle to bovine tuberculosis [68]. Furthermore, *CXCL10* has previously been reported to be up-regulated in the bovine immune response to several pathogenic species [30] including *Eimera bovis* [69, 70], *Toxoplasma gondii* [65], *Neospora canium* [65], Alcelaphine herpesvirus 1 [59], bovine respiratory syncytial virus [71] and *Mycobacterium bovis* [66]. The *GZMA* gene has been observed to be transcriptionally increased in response to viral challenge with both bovine herpesvirus 1 and bovine respiratory syncytial virus [71]. Furthermore, *PDGFRA* transcription has increased in response to viral challenges with both bovine respiratory syncytial virus and bovine viral diarrhoea virus and in response to bacterial challenge with *Mannheimia haemolytica* and *Pasteurella multocida* [71]. Increased expression of *TNFSF12* was observed following infection with both bovine herpesvirus 1 and bovine viral diarrhoea virus [71]. The *CXCL4* cytokine has been up-regulated in response to a challenge infection with *Mannheimia haemolytica* [71]. Transcription of *CALCRL* has been increased in response to viral challenge with bovine viral diarrhoea virus and bacterial challenge with *Mannheimia haemolytica* [71]. Finally, *LTB4R* transcription increased following challenge infections with bovine viral diarrhoea virus, bovine herpesvirus 1 and *Pasteurella multocida* [71].

This study has demonstrated that H-F calves have consistently higher basal levels of transcription of many cytokines, chemokines and immune response effector genes, than J calves, under artificial-rearing conditions. Higher basal levels of pro-inflammatory cytokines may also infer better resistance to pathogens in the environment as Li and colleagues [67] have shown that heifers resistant to nematode infections are better able to produce inflammatory cytokines at the site of nematode infection than susceptible heifers. Therefore, the increased levels of transcription of many immune response associated genes in H-F calves suggests that this breed may be better equipped to fight pathogen invasion more rapidly before pathogens can cause disease. Indeed, we have observed J calves to succumb to greater numbers of incidents of bovine respiratory disease, than H-F calves (unpublished data). Furthermore, as the many of the DEG which were decreased in J relative to H-F calves were involved in natural killer cell and Th 1 cell activity which primarily function in the clearance of intracellular bacteria and virus infected cells [35, 72], J calves may specifically be more susceptible than H-F calves to infection with intracellular bacteria and some viruses. Indeed, J have been reported to develop infections with *Mycobacterium paratuberculosis* more often than H-F [73, 74] and J calves have been found to be more susceptible to *Salmonella typhimurium* infection than Friesian calves [75].

The decreased transcription of genes involved in immune responses in J relative to H-F calves was, however, surprizing as the J calves in the present study, along with J calves in other studies [15, 76], have higher maternally derived serum immunoglobulin G concentrations. It appears that despite J calves receiving enhanced immunological protection from disease from their dams, they still may have reduced immune capacity. Although greater transcription of pro-inflammatory cytokines in H-F relative to J calves may improve efficiency of both the immune responses to pathogens and resistance to infection, it is also possible that they may cause pathogenesis. When unregulated excess pro-inflammatory chemokines are produced, they can cause tissue damage and exuberate many inflammatory diseases such as allergic reactions, arthritis and arteriosclerosis [31, 34, 77].

Differential gene expression between MAP experimentally challenged Holstein and J cows has been already demonstrated in a microarray experiment [14] and we have shown in the present study, differential gene expression between experimentally unchallenged H-F and J calves. Furthermore, using pathway analysis tools, we have predicted differences in functional immune responses, including leukocyte chemotaxis and phagocytosis. Interestingly, whole blood killing of bacteria and neutrophil oxidative burst, following incubation of blood with enteropathogenic *Escherichia coli*, have previously been demonstrated to be decreased in J relative to Holstein calves [15] and this is in agreement with predicted immune function differences between the breeds using the DEG in our study. Despite these observations, only minimal breed differences between the innate immune responses of Holstein and J cows to experimental challenge intramammary infection with both *Staphylococcus aureus* [78] and *Escherichia coli* [13] have been reported. However, we have discovered decreased transcription in J relative to H-F calves, in genes which promote cytotoxic activity in natural killer cells and T lymphocytes. Consequently, future experimental challenge studies comparing H-F and J immune responses to disease could be designed using either an intracellular bacterium or a virus and subsequently, immune functional tests could be carried out on blood collected from these animals during the experimental challenge.

The H-F and J calves in this study were, by necessity, sourced from different herds. Consequently, there was in a difference in age at study enrolment between the H-F and J calves. However, both age at allocation to treatment and serum ZST at arrival on site, were included as co-variants in the statistical models in the larger study, Johnston, *et al*. [8], and both were found to be statistically non-significant. Therefore, it is our contention that the initial minor variation in age between the breeds at the start of the study did not have any confounding effect on the interpretation of our results.

## Conclusions

These results demonstrate that gradual weaning is welfare friendly as it does not induce transcriptional alterations during the peri-weaning period. Therefore, the necessary husbandry practice of weaning potentially has minimal negative effects on the welfare and health of dairy calves if it is carried out gradually when calves are consuming sufficient concentrate feed and consequently have rumens which are sufficiently developed to derive adequate nutrients from solid feed alone. Additionally, these data also demonstrate differences in transcriptional activity of genes involved in immune responses and cell signalling activity between the two breeds. They suggest decreased cellular movement, chemotaxis and phagocytic functionality in J relative to H-F calves. Knowledge of breed-specific immune responses could enable improved health management practices tailored towards H-F and J specific disease sensitivities. This knowledge could also promote and enable development of cost effective breed targeted prophylactic and therapeutic veterinary interventions.

## Supporting Information

S1 Table Differentially expressed genes between Holstein-Friesian and Jersey calves at day -14, relative to weaning (P < 0.05; FDR < 0.1; fold change > 0.05). (XLSX)

S2 Table Differentially expressed genes between Holstein-Friesian and Jersey calves at day 1, relative to weaning (P < 0.05; FDR < 0.1; fold change > 0.05). (XLSX)

S3 Table Differentially expressed genes between Holstein-Friesian and Jersey calves at day 8, relative to weaning (P < 0.05; FDR < 0.1; fold change > 0.05). (XLSX)

S4 Table The molecular functions, biological processes and cellular components which were over-represented among the DEG between Holstein-Friesian and Jersey calves, at each time-point relative to weaning, using the GoSeq analysis. (XLSX)

S5 Table Functional annotation clusters identified among the DEG between Holstein-Friesian and Jersey calves at day -14, relative to weaning. Enriched functional annotation clusters (group enrichment score > 1.3) are highlighted in yellow. Functional annotations within the clusters which had a benjamini hochberg false discovery rate < 0.1 are highlighted in blue.
(XLSX)

S6 Table Functional annotation clusters identified among the DEG between Holstein-Friesian and Jersey calves at day 1, relative to weaning. Enriched functional annotation clusters (group enrichment score > 1.3) are highlighted in yellow. Functional annotations within the clusters which had a benjamini hochberg false discovery rate < 0.1 are highlighted in blue.
(XLSX)

S7 Table Functional annotation clusters identified among the DEG between Holstein-Friesian and Jersey calves at day 8, relative to weaning. Enriched functional annotation clusters (group enrichment score > 1.3) are highlighted in yellow. Functional annotations within the clusters which had a benjamini hochberg false discovery rate < 0.1 are highlighted in blue.
(XLSX)

S8 Table Gene functional classification groups identified among the DEG between Holstein-Friesian and Jersey calves at day -14, relative to weaning. Enriched gene functional classification groups (group enrichment score > 1.3) are highlighted in yellow.
(XLSX)

S9 Table Gene functional classification groups identified among the DEG between Holstein-Friesian and Jersey calves at day 1, relative to weaning. Enriched gene functional classification groups (group enrichment score > 1.3) are highlighted in yellow.
(XLSX)

S10 Table Gene functional classification groups identified among the DEG between Holstein-Friesian and Jersey calves at day 8, relative to weaning. Enriched gene functional classification groups (group enrichment score > 1.3) are highlighted in yellow.
(XLSX)

S11 Table Predicted activation states (increased or decreased) of biological functions identified by Ingenuity Pathway Analysis as enriched among the DEG between Holstein-Friesian and Jersey calves at day -14, relative to weaning. Genes coloured in red have increased expression in Jersey relative to Holstein-Friesian calves. Genes coloured in green have decreased expression in Jersey relative to Holstein-Friesian calves.
(XLSX)

S12 Table Predicted activation states (increased or decreased) of biological functions identified by Ingenuity Pathway Analysis as enriched among the DEG between Holstein-Friesian and Jersey calves at day 1, relative to weaning. Genes coloured in red have increased expression in Jersey relative to Holstein-Friesian calves. Genes coloured in green have decreased expression in Jersey relative to Holstein-Friesian calves.
(XLSX)

S13 Table Predicted activation states (increased or decreased) of biological functions identified by Ingenuity Pathway Analysis as enriched among the DEG between Holstein-Friesian and Jersey calves at day 8, relative to weaning. Genes coloured in red have increased expression in Jersey relative to Holstein-Friesian calves. Genes coloured in green have decreased expression in Jersey relative to Holstein-Friesian calves.
(XLSX)
